# Characterization of SARS-CoV-2 ORF6 deletion variants detected in a nosocomial cluster during routine genomic surveillance, Lyon, France

**DOI:** 10.1080/22221751.2021.1872351

**Published:** 2021-01-27

**Authors:** Grégory Quéromès, Grégory Destras, Antonin Bal, Hadrien Regue, Gwendolyne Burfin, Solenne Brun, Rémi Fanget, Florence Morfin, Martine Valette, Sophie Trouillet-Assant, Bruno Lina, Emilie Frobert, Laurence Josset

**Affiliations:** a CIRI, Centre International de Recherche en Infectiologie, Team VirPatH, Lyon, Fracne; b Laboratoire de Virologie, Institut des Agents Infectieux (IAI), Hospices Civils de Lyon, Lyon, France; c Centre National de Référence des virus des infections respiratoires, Lyon, Fracne

**Keywords:** SARS-CoV-2, ORF6, deletion, genomic surveillance, nosocomial cluster, inflammation

## Abstract

During routine molecular surveillance of SARS-CoV-2 performed at the National Reference Center of Respiratory Viruses (Lyon, France) (*n* = 229 sequences collected February–April 2020), two frameshifting deletions were detected in the open reading frame 6, at the same position (27267). While a 26-nucleotide deletion variant (D26) was only found in one nasopharyngeal sample in March 2020, the 34-nucleotide deletion (D34) was found within a single geriatric hospital unit in 5/9 patients and one health care worker in April 2020. Phylogeny analysis strongly suggested a nosocomial transmission of D34, with potential fecal transmission, as also identified in a stool sample. No difference in disease severity was observed between patients hospitalized in the geriatric unit infected with WT or D34. *In vitro* D26 and D34 characterization revealed comparable replication kinetics with the wild-type (WT), but differential host immune responses. While interferon-stimulated genes were similarly upregulated after infection with WT and ORF6 variants, the latter specifically induced overexpression of 9 genes coding for inflammatory cytokines in the NF-kB pathway, including *CCL2/MCP1*, *PTX3*, and *TNFα*, for which high plasma levels have been associated with severe COVID-19. Our findings emphasize the need to monitor the occurrence of ORF6 deletions and assess their impact on the host immune response.

## Introduction

The coronavirus disease 2019 (COVID-19) pandemic triggered by the novel severe acute respiratory syndrome coronavirus 2 (SARS-CoV-2) virus has continued to spread globally since its emergence in China in late 2019 [1,2]. Countries and localities have implemented various levels of public health mitigation measures with debatable success in an effort to control virus propagation [3–6]. The challenge in better understanding the fundamental characteristics of this novel virus includes the heterogeneous disease reports in conjunction with no clear treatments or vaccines yet available or approved [7–9]. Epidemiological tracking is paramount in the context of this current pandemic [10,11]. In particular, the genomic surveillance of circulating virus variants, such as with the seasonal epidemics of the influenza virus or even with the 2003 SARS epidemic, has brought useful information in understanding their respective evolutionary dynamics [12,13]. Recent tracking reports have discussed the high frequency and global distribution of a variant harbouring the D614G substitution located on the SARS-CoV-2 spike protein [14]. While higher infectious titre and increased protein stability have been associated with this variant, a clear fitness advantage has not been unequivocally established [15,16]. Historically, evolution of the related SARS-CoV virus is defined by deletion regions that impact the open-reading frames (ORF) of its genome [17,18]. Several deletions of large variations in size and prevalence have already been described in the SARS-CoV-2 genome [19–22].

The aim of this study was to therefore describe clinical patient data, viral replication capacity, and host innate immune modulation of two newly detected ORF6 deletion variants detected in early April from routine genomic surveillance of COVID-19 patients in Lyon, France.

## Material and methods

### Sequencing

Early routine genomic surveillance of SARS-CoV-2 in the National Reference Center (NRC) of Respiratory Viruses is based on daily random selection of samples with SARS-CoV-2 detected with quantitative reverse-transcriptase polymerase chain reaction (qRT-PCR) cycle threshold (Ct) <20 [6], which were then sequenced using an RNA metagenomic next-generation sequencing (mNGS) method previously described [19]. Briefly, viral genetic material contained in nasopharyngeal and stool samples was extracted by the EMAG® platform (bioMerieux, Lyon, FR). After DNAse treatment (Life Technologies, Carlsbad, CA, USA), samples underwent random amplification using Whole Transcriptome Amplification (WTA2 kit, Sigma-Aldrich, Darmstadt, DE) before sequencing on an Illumina NextSeqTM 550 with mid-output 2 × 150 flow cell. Importantly, the variants displaying an ORF6 deletion were confirmed by 3 other techniques, including capture- and amplicon-based strategies [23]. Sequencing of patient samples began on February 8th and is ongoing. For the stool sample, an amplicon-based approach developed by the ARTIC network (https://artic.network/ncov-2019) combined with Oxford Nanopore Technologies sequencing was used.

### Phylogeny

Multiple sequence alignment was performed using the DECIPHER package in R [24]. Pairwise distances were computed using the Kimura (K80) model implemented in the function dist.dna, deleting the sites with missing data in a pairwise way. The phylogenetic tree was constructed using R software using ape package and the neighbour joining evolutionary method (hCoV19/Wuhan/IPBCAMSWH01/2019 as the root). CoV-GLUE resource [http://cov-glue.cvr.gla.ac.uk, [25]] was used to generate phylogenetic placement of the mutants, annotate the sequences, and check the prevalence of the deletions among worldwide sequences. Codon numbering is based on the Wuhan-Hu-1 sequence.

### Virus replication kinetics

Replication kinetics was performed on both confluent buffalo green monkey (BGM) (BioWhittaker Europe) and human lung adenocarcinoma (CaLu-3) cells (ATCC® HTB-55™, Plateforme iPS, NeuroMyoGene Institute, Lyon, FR, [26]) at a multiplicity of infection (MOI) of 10^−3^ at 36°C with 5% CO_2_ for 7 days, fully respecting the WHO interim biosafety guidance related to the coronavirus disease [27]. Comparative viral particle quantification of culture supernatant was performed by RdRp Institut Pasteur qRT-PCR on a QuantStudio™ 5 System (Applied Biosystems, ThermoFisher Scientific, MA, USA) with a standard curve after semi-automated EMAG® extraction (bioMérieux, Lyon, FR) [6]. Statistical analysis was performed by two-way ANOVA with Tukey multiple comparisons between both factors of comparison (virus variant and cell line) on GraphPad Prism (software version 8.4.3).

### Immune-related gene expression profiling

Confluent CaLu-3 cells were inoculated in triplicate with wild-type or ORF6 deletion strains at 0.2 MOI and incubated for 24 h at 36°C with 5% CO_2_. For basal transcriptomic levels, a mock infection condition was also tested in triplicate. Cellular RNA extraction was performed with the RNeasy Mini Kit (Qiagen, DE) after supernatant removal and cell lysis directly in the culture vessel. Purified RNA was quantified with the Qubit RNA HS Kit (ThermoFisher Scientific, MA, USA). Host gene expression was evaluated using an 87-gene panel (Supplementary Table 1) with NanoString technology. Data treatment was performed using nSolver analysis software (version 4.0, NanoString Technologies). To normalize for differences in RNA input we used the geometric means over four housekeeping genes (*DECR1, HPRT1*, *RPL19*, and *RPLP0*).

Finally, the log_2_ fold change (log_2_ FC) between the infection conditions and the mock infection control were calculated to evaluate the transcriptomic modifications induced by SARS-CoV-2 strains. Criteria for differential expression were an absolute log_2_ FC of 1 and a *q*-value < 0.05 calculated using a Students’ t-test with subsequent Benjamini-Hochberg correction.

### Ethics

Samples used in this study were collected as part of an approved ongoing surveillance conducted by the National Reference Center for Respiratory Viruses (NRC) in France (WHO reference laboratory providing confirmatory testing for COVID-19). The investigations were carried out in accordance with the General Data Protection Regulation (Regulation (EU) 2016/679 and Directive 95/46/EC) and the French data protection law (Law 78–17 on 06 January 1978 and Décret 2019–536 on 29 May 2019). Samples were collected for regular clinical management during hospital stay, with no additional samples for the purpose of this study. Patients were informed of the research and their non-objection approval was confirmed. This study was presented by the ethics committee of the Hospices Civils de Lyon (HCL), Lyon, France and registered on the HCL database of RIPHN studies (AGORA N°41).

### Data availability

The SARS-CoV-2 genomes sequenced in this study were deposited on the GISAID database (https://www.gisaid.org/) on a regular basis, accession numbers can be found in Supplementary Table 2.

## Results

### ORF6 deletion variants detected during routine genomic surveillance

As part of the Auvergne-Rhône-Alpes (ARA) regional surveillance, 229 samples collected between 2 February and 12 April 2020 were sequenced by the French National Reference Center of Respiratory Viruses. These samples originated mainly from the Hospices Civils de Lyon (HCL) (149 sequences from 58 units within 11 different hospital sites), with some from other hospitals in the Lyon area (24 sequences) and other regional hospitals (56 sequences, 12 cities).

**Figure 1.**
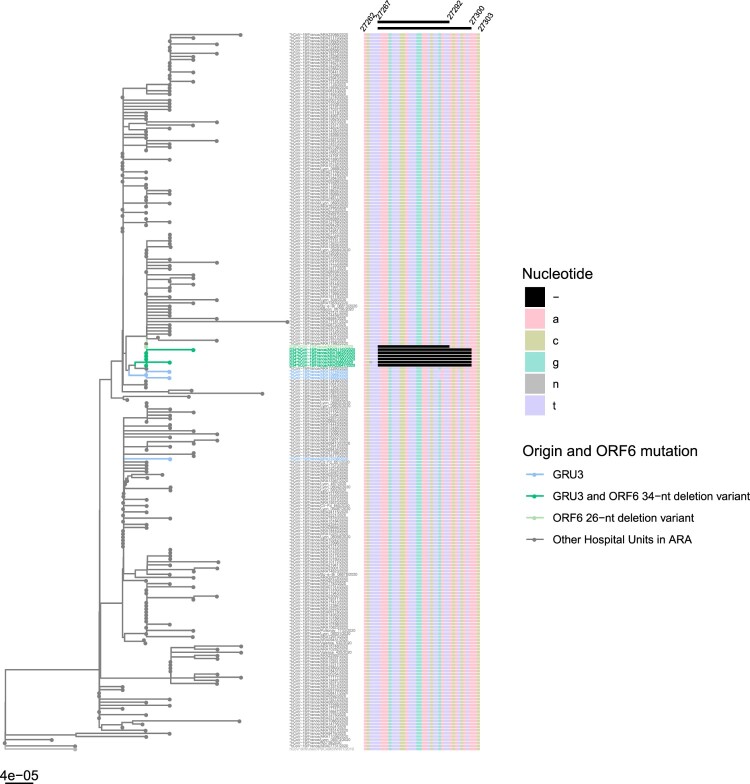
Phylogenetic tree of SARS-CoV-2 full genome sequences from Auvergne-Rhône-Alpes (ARA) patients (*n* = 229). The phylogenetic tree was constructed using R software with ape package and the neighbour joining evolutionary method (hCoV-19/Wuhan/IPBCAMSWH01/2019 (EPI_ISL_402123) as the root). The coloured branches denote the hospital unit origin of the sequence and ORF6 status. On the right, multiple sequence alignment from nucleotide position 27267–27303 (Wuhan-Hu-1 numbering) is illustrated, with the 26-nt and 34-nt deletions depicted in black. The deletion sites of interest were not included for genetic distance calculation. GISAID accession number for the four GRU3 WT (EPI_ISL_508986, EPI_ISL_418431, EPI_ISL_508987, EPI_ISL_419180), six GRU3 D34 sequences (EPI_ISL_508992, EPI_ISL_508971, EPI_ISL_508920, EPI_ISL_508989, EPI_ISL_508988, EPI_ISL_508919) and one D26 sequence (EPI_ISL_508941). GRU = geriatric rehabilitation unit.

**Figure 2.**
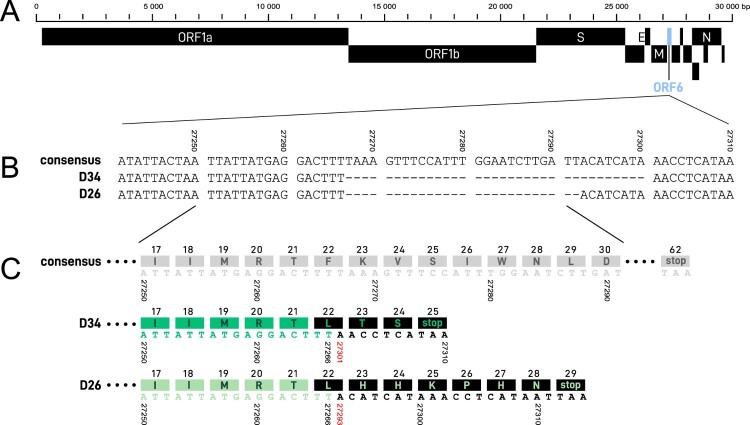
(A) Complete SARS-CoV-2 genome map with ORF6 illustrated in blue. (B) Nucleotide sequences at consensus ORF6 site 27241-27310. Both 34-nt (D34) and 26-nt (D26) deletion regions, beginning at position 27267, are denoted by dashes. (C) Partial ORF6 region at consensus nucleotide position 27250–27291 with corresponding amino acids depicted in the coloured blocks above the nucleic acid sequence. Nucleic acids and amino acids are illustrated in gray for the consensus sequence, in green for the deletion variant sequences, and in black for the alterations resulting from the ORF6 deletions, with presumptive premature stop codons.

Of these 229 samples, 6 sequences were shown to carry a 34-nt deletion (at position 27267–27300), henceforth denoted as D34, and 1 sequence a 26-nt deletion (at position 27267–27292), denoted as D26 (Figure 1). These deletions are both frameshifting deletions in the ORF6, starting at the same 27267 position after a stretch of 3 T at 27264–27266 (Figure 2). Because of the frameshifting, the D34 variant generates a premature stop codon (at position 27308, Wuhan-Hu-1 numbering), resulting in a presumed truncated 24 amino acid protein, instead of 61 in the wild-type (Wuhan-Hu-1 and all other sequences described yet). The D26 variant yields a 28 amino acid protein with its premature stop codon at position 27312 (Wuhan-Hu-1 numbering) (Figure 2(C)). These deletions have not yet been described on the CoV-GLUE resource, which lists all genomic variants in SARS-CoV-2 sequences available on the GISAID database (Supplementary Figure 1). Of note, D26 is annotated at different positions in CoV-GLUE to maximize amino acid alignment (27265–27290).

The 7 sequences carrying an ORF6 deletion belong to lineage B1, a lineage widely circulating in Europe (Supplementary Figure 1). There were between 0 and 3 SNP (Single Nucleotide Polymorphism) differences among D34 variants, for which 3/6 mutants displayed 1–3 SNPs, and between 2 and 4 SNPs between D26 and D34 variants (Supplementary Figure 2).

### Evidence for direct transmission of the ORF6 34-nt deletion variant

The D34 samples were all collected from hospitalized patients or health care workers (HCW) in the same geriatric rehabilitation unit in the Hospices Civils de Lyon (GRU-3), between April 2nd and April 9th, while the sample with the 26-nt deletion was collected one month earlier (March 10th) in a geriatric unit of another hospital (Table 1). The hospitals are 80 km apart and there was no evidence for the transfer of patient #73 with the 26-nt deletion into GRU-3. To track the origin of the deletion, all patients hospitalized in the GRU-3 geriatric unit and all samples collected between March 18th and April 9th with high viral loads of SARS-CoV-2 (RT–PCR Ct value < 20) were sequenced (*n* = 9). In total, 44% (4/9 patients) were infected with the WT SARS-CoV-2 ORF6 (samples collected between March 18th–30th), with no read carrying the deletion at a minor frequency. Out of the 4 WT SARS-CoV-2 ORF6, three sequences were very similar to D34 and carried a G27289 T SNP (D30Y), which has already been identified in three patients from England between April 20th and 27th, 2020 (Figure 1). The other 55% (5/9 patients), in addition to 1 HCW, were infected with the 34-nt deletion (samples collected after April 2nd) with 100% of the reads carrying the deletion for each patient. Overall, 8/9 sequences of GRU-3, corresponding to those of the D34 variants and those of the three WT strains carrying the ORF6 G27289 T SNP were clustered together, while the sequence of the patient #38 was more divergent. We could not investigate whether the mutation spread within this unit after April 9th as only one COVID-19 patient was hospitalized in this geriatric unit since, for which their viral load (Ct > 30) was too low for mNGS.

**Table 1.** Clinical data from patients infected with the D34 ORF6 deletion variant compared with related patients infected with WT SARS-CoV-2.

	Patient #68	Patient #38	Patient #46	Patient #65	Patient #25	Patient #8	HCW #63	Patient #60	Patient #47	Patient #50
**Sequence name**^a^	ARA10968/2020	ARA12238/2020	ARA13746/2020	ARA16665/2020	ARA18625/2020	ARA2068/2020	ARA21163/2020	ARA21360/2020	ARA22647/2020	ARA22650/2020
**Age (years)**	89	78	84	89	97	82	31	94	81	85
**Sex**	F	M	F	F	M	F	F	F	M	F
**Comorbidities**	Hypertension	None	None	Cardiovascular disease, Hypertension	Cardiovascular disease, Hypertension	Cardiovascular disease, Hypertension, Obesity	NA	Cardiovascular disease, Hypertension	None	Cardiovascular disease
**Respiratory manifestations**	URTI	LRTI	LRTI	Asymptomatic	Asymptomatic	LRTI	URTI	URTI	Not specified	LRTI
**Hospitalization (Yes / No)**	Yes	Yes	Yes	Yes	Yes	Yes	No	Yes	Yes	Yes
**Hospitalization Unit**	GRU-3	GRU-3	GRU-3	GRU-3	GRU-3	GRU-3	GRU-3	GRU-3	GRU-3	GRU-3
**ORF6 deletion**	None	None	None	None	D34	D34	D34	D34	D34	D34
**Date of diagnosis**	2020/03/18	2020/03/22	2020/03/25	2020/03/30	2020/04/02	2020/04/06	2020/04/07	2020/04/07	2020/04/09	2020/04/09
**Duration of excretion**^b^	Respiratory sample positive at D21	Not monitored	Not monitored	Respiratory sample positive at D14	Not monitored	Not monitored	Not monitored	Not monitored	Respiratory and stool samples positive at D14	Not monitored
**Outcome**^b^	Favorable	Favorable	Favorable	Favorable	Deceased, unrelated to COVID-19 (D5)	Deceased from COVID-19 (D6)	Favorable	Favorable	Favorable	Deceased from COVID-19 (D9)

HCW = health care worker; COPD = chronic obstructive pulmonary disease; URTI: Upper Respiratory Tract Infection; LRTI: Lower Respiratory Tract Infection; HCL = Hospices Civils de Lyon; GRU-3 = geriatric rehabilitation unit within HCL; D26 = 26-nt deletion; D34 = 34-nt deletion; D = Day.

^a^ Sequence names in the table are preceded by hCoV-19/France/; example: hCoV-19/France/ARA10968/2020.

^b^ Duration of excretion and death are calculated from the date of diagnosis.

### 34-nt ORF6 deletion variants yielded similar clinical presentations as WT ORF6 in hospitalized patients

Clinical data were studied on hospitalized patients in GRU-3 (i.e. excluding the HCW #63 to better control for confounding variables (e.g. age and comorbidities)) to compare COVID-19 severity between patients infected with D34 and with WT SARS-CoV-2 (*n* = 9) (Table 1). The median age of hospitalized patients was 87 years (ranging from 78 to 97), with 7 patients presenting at least cardiovascular disease as a risk factor (Table 1). Other comorbidities included hypertension (*n* = 5), obesity (*n* = 1), and chronic obstructive pulmonary disease (*n* = 1).

Clinical presentations of hospitalized patients with the D34 variants (*n* = 5) were classified as asymptomatic for one patient, upper respiratory tract infection (URTI) for 2 patients, and lower respiratory tract infection (LRTI, pneumonia) for 3 patients. To evaluate disease severity in relation to the D34 deletion, mild (asymptomatic and URTI, *n* = 5) versus severe COVID-19 (LRTI, *n* = 4) was compared by Fisher’s exact test. No significant difference in clinical presentation could be observed between hospitalized patients harbouring or not the ORF6 deletion (*p* > 0.99).

From the five hospitalized patients harbouring D34 deletion, 2 died from COVID-19 infection, all presenting LRTI and comorbidities. One patient (#25) died at day 5 after diagnosis, but their death was not related to COVID-19 infection but to septicemia. To evaluate disease outcome in relation to the D34 deletion, death from COVID-19 versus favorable outcome (including non-COVID-19 death) was compared by Fisher’s exact test. No significant difference in disease outcome could be observed between hospitalized patients harbouring or not the D34 deletion (*p* = 0.44).

Notably, patient #47 harbouring a D34 variant was still positive after 14 days in respiratory and stool samples. Virus present in the stool was 100% identical to the first virus sequenced from respiratory samples. Unfortunately, the respiratory sample at day 14 could not be sequenced due to Ct > 30.

### SARS-CoV-2 deletion variants yield comparable replication kinetics to reference strain

Two genomes representative of ORF6 deletion variants found in this regional circulation were selected for replication tests: hCoV-19/France/ARA22647/2020 (EPI_ISL_508919; D34 variant) and hCoV-19/France/ARA0731/2020 (EPI_ISL_508941; D26 variant). These genomes were compared against the reference genome hCoV-19/France/ARA24023/2020 (EPI_ISL_508931; devoid of any deletions), which was the most similar isolated variant sequenced available in the laboratory at the time of the investigation. The reference genome had 1–3 SNPs compared with D26 and D34 variants (Supplementary Figure 2).

**Figure 3.**
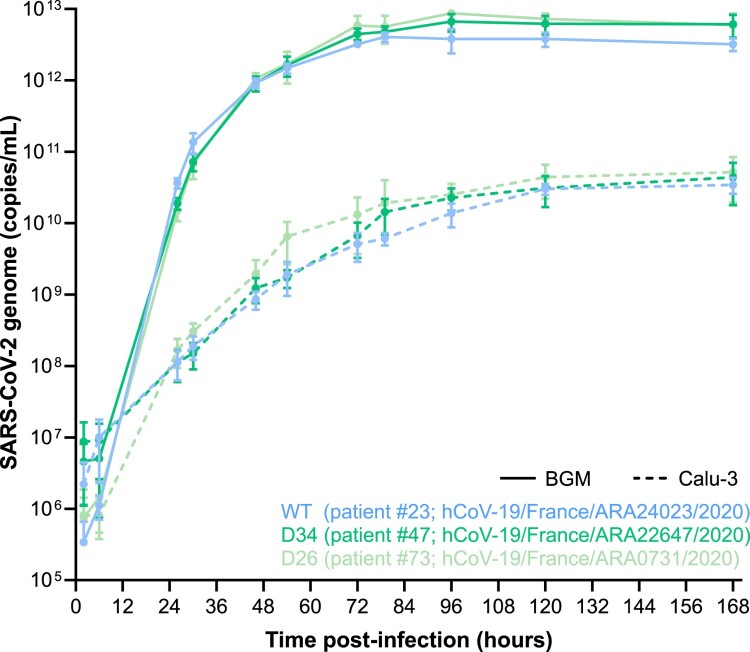
Replication kinetics of SARS-CoV-2 ORF6 deletion variants on BGM and CaLu-3 cell lines. SARS-CoV-2 variants were inoculated on confluent BGM and CaLu-3 cells at an MOI of 10^−3^ and then incubated at 36°C with 5% CO_2_ for 7 days. Supernatant samples were collected at regular intervals, for which viral particle quantification was performed by qRT-PCR. Each data point is the average of three replicates, with standard deviation as error bars. Statistical analysis was performed by two-way ANOVA with Tukey multiple comparisons between both factors of comparison (virus variant and cell line) on GraphPad Prism (software version 8.4.3).

Replication kinetics measured by viral genome quantification revealed no significant difference between the three variants throughout the course of *in vitro* infection on both BGM and CaLu-3 cell lines (Figure 3). However, a significant difference was observed between cell lines for each variant, with an increased level of replication on BGM (as early as 24 h post-inoculation). More specifically, viral replication spiked rapidly on BGM cells within the first 48 h, before reaching a plateau at 72 h. Conversely, viral replication on CaLu-3 cells rose steadily within the first 48 h, before reaching a plateau at 96 h. Of interest, a 2-log difference was observed for maximum genome quantification between BGM and CaLu-3, with an average of 5.76 × 10^12^ and 4.01 × 10^10^ copies/mL, respectively.

### Differential immune gene expression from ORF6 deletion SARS-CoV-2 infection

**Figure 4.**
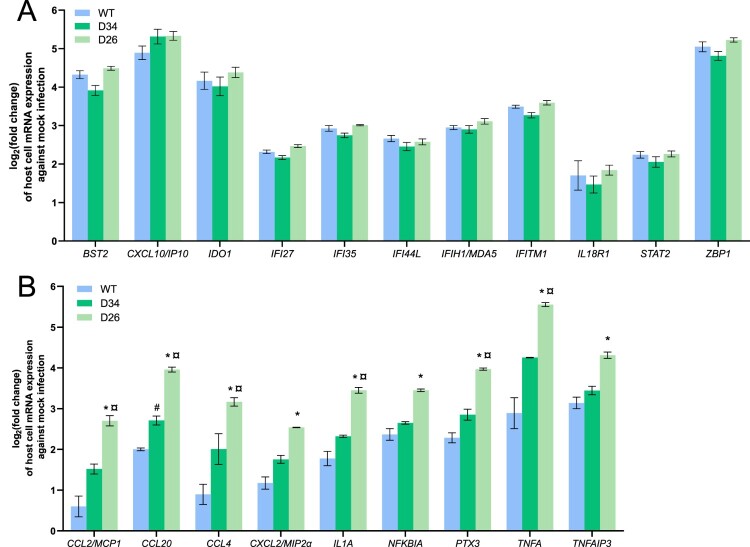
*In vitro* host immune gene expression by NanoString profiling 24 h post-infection of WT or ORF6 deletion SARS-CoV-2 variants on CaLu-3 cells. (A) Host cell transcriptomics profile of immune-related genes with highest log_2_Fold Change (FC) against mock infection yielding no significant difference between WT and ORF6 deletion infections. (B) Differential host cell transcriptomics profile of immune-related genes between ORF6 deletion infections and WT infection. D34 = 34-nt ORF6 deletion; D26 = 26-nt ORF6 deletion. *significant difference between D26 and WT (*q* < 0.05 and log_2_FC > 1); # significant difference between D34 and WT (*q* < 0.05 but log_2_FC < 1); ¤ significant difference between D34 and D26 (*q* < 0.05 and log_2_FC > 1).

As ORF6 codes for a protein involved in innate immunity regulation, we aimed to explore the impact of D34 and D26 on the immune transcriptome at 24 h post infection. Of the 87 human host immune genes tested on the Nanostring Immunity panel, 40 were significantly upregulated following infection by WT SARS-CoV-2 compared to the mock infection on CaLu-3 cells (Supplementary Figure 3, Supplementary Table 2). These upregulated genes belong to the following pathways: inflammation mediation by chemokine and cytokine signalling, interleukin signalling, toll receptor signalling, and apoptosis signalling. *BST2*, *CXCL10/IP10*, *IDO1*, and *ZBP1* presented the highest upregulation with 4.32, 4.89, 4.16, and 5.05 log_2_FC, respectively (Figure 4(A)). Interestingly, there was no difference of their expression with the deletion variants, as well as for certain interferon-stimulated genes (ISG), including *IFI27*, *IFI35*, *IFI44L*, *IFIH1/MDA5*, *IFITM1*, *IL18R1*, and *STAT2* (Figure 4(A)). However, an enhanced upregulation of 9 genes, all involved in the NF-kB pathway (*CCL2/MCP1*, *CCL20*, *CCL4*, *CXCL2/MIP2α*, *IL1A*, *NFKBIA*, *PTX3*, *TNFA*, *TNFAIP3*), was observed after infection with D26 variants, in comparison to the WT (Figure 4(B)). While higher levels of expression of these cytokines were also noted after infection with the D34 variant compared with WT, increased expression did not reach statistical significance after multi-test correction (q-value < 0.05), with the exception of *CCL20*. Of note, the D26 variant induced a markedly higher level of expression for 6 of these 9 genes (*CCL2/MCP1*, *CCL20*, *CCL4*, *IL1A*, *PTX3*, *TNFA*) than the D34 variant. This difference was not due to higher replication of D26, as equivalent viral quantification was observed at 24 h for all conditions, with an average of 7.94 log_10_ copies/mL. Altogether, these results show that D26 and D34 do not impact ISG expression but may increase NFKB-driven inflammatory genes, and with a higher magnitude for D26.

## Discussion

Despite reports of the relative stability of the SARS-CoV-2 genome within the human population, whole genome sequencing has revealed recurrent variants with variable mutation patterns over the course of the pandemic and within distinct geographic regions [10,19,28–31]. Here, we characterized large ORF6 deletion variants identified through surveillance of patients from the same hospital unit. Reports of similar patterns of genomic deletions in the SARS-CoV-2 genome since its emergence have already been described, including in the ORFs 6, 7, and 8 [20,21,29,32].

The origin of the D34 deletion is still unknown. However, as the WT virus isolated from GRU-3 patients were genetically close to the D34 variants, the GRU-3 patients infected by the WT virus in March could have been the initial source of the D34 deletion. None of the GRU-3 patients with WT or deletion variants presented any intra-host diversity in the ORF6 deletion region that would have been indicative of a recent mutation or recombination. Nevertheless, the D34 variants’ introduction since April 2nd with its limited presence in the hospital unit thereafter and the clustering of the corresponding whole genome sequences by phylogeny analysis strongly suggest a nosocomial transmission of the D34 variant. Importantly, the persistence of the same D34 consensus sequence in a patient’s stool sample 14 days after diagnosis from a nasopharyngeal sample gives emphasis to the enteric tropism capacity of D34 variants and the potential contribution of fecal transmission to nosocomial transmission [33].

The normal rate of mutation of SARS-CoV-2 has been reported at about 2.5 mutations per month [34,35]. Given that D34 variants had 1–3 SNP differences between consensus sequences of D34 variants collected between one week, a higher mutation rate than normal might be linked to adaptative mutations following the deletion. During the revisions of the manuscript, 3 novel sequences of the D34 variant sampled in May at the university hospital of Lyon (EPI_ISL_683361; EPI_ISL_683360; EPI_ISL_683354) and 1 sequence of the D26 variant sampled on April 23rd in a hospital located 75 km from Lyon (EPI_ISL_660432) were detected out of the 932 viruses sequenced to date in our laboratory. This suggests a relatively limited diffusion of these variants. Nevertheless, the importance of such genomic deletion variants by NGS investigation during the evolution of disease transmission and population prevalence should not be overlooked [13,36,37]. Evidence of adaptation by means of genomic deletions during the middle and late phases of the SARS-CoV 2003 epidemic has been tenuously described [17,18,38–40].

As research on the SARS-CoV ORF6 has attributed this accessory protein (p6) with potential functions of intracellular membrane rearrangements, of interferon induction inhibition, and of replication stimulation [41–43], we performed a global *in vitro* characterization of the ORF6 variants. Firstly, no significant impact was noted on replication capacity *in vitro* in comparison to a wild-type strain, in two different cell lineages. The comparable replication kinetics between wild-type and deletion variants determined *in vitro* is supported by the congruent *in vivo* replication capacity with the latter being assessed by RT–PCR from diagnosis (Ct < 20).

Secondly, we investigated whether the ORF6 variants could modulate innate immune responses. The important upregulation of certain genes induced by a SARS-CoV-2 infection observed in the present study were in accordance with typical antiviral restriction responses, such as *BST2*, *IDO,* and *IFTM1* [44–46]. Focusing on the two deletion variants, the expression of interferon-stimulated genes (ISG) was not differentially expressed in comparison with the WT virus, suggesting that the resulted truncated proteins from the two ORF6 deletions did not impact this signalling pathway. This is in contrast to previous findings of interferon pathway dysregulation via STAT1/STAT2 nuclear translocation blocking by the p6 protein [47] Nevertheless, our transcriptomic analyses revealed an enhanced upregulation of 9 immune-related genes in the NF-kB pathway, including those coding for inflammatory cytokines such as chemokines, IL1A and TNFα, specifically induced by the infection of partial ORF6 deletions. These findings suggest that the p6 protein would interact in an antagonistic manner to suppress their antiviral properties. Recent literature confirms this inhibitory function of the SARS-CoV-2 p6 protein [48–50]. Of interest, the D26 variant, which resulted in a longer protein sequence *in silico* than for the D34 variant, presented an even higher upregulation of these NFKB-driven inflammatory genes. It can be hypothesized that D26 yields a complete loss of function of p6, perhaps due to the additional histidine residues in close proximity to the α-helix, while D34 may lead to a p6 with a somewhat conserved activity. A 27-nt in-frame ORF6 deletion in proximity to D34 and D26 (at position 27264–27290) selected during passaging on Vero6 cells had important three-dimensional structural alterations to the protein [32].

Most importantly, high plasma levels of PTX3, MCP1, and TNFα in COVID-19 patients have been described as early molecular indicators of adverse disease progression needing intensive care [7,51]. In addition, within the context of cytokine storms from acute respiratory syndromes, high plasma levels of CCL2, CXCL10, and TNFα have also been reported [48,52]. Consequently, the enhanced expression of *CCL2/MCP1*, *PTX3* and *TNF*α observed after infection with D26 could indicate a heightened disease risk from an NF-kB-driven inflammatory response by an ORF6 deletion variant [53]. Although there was no significant difference in disease severity between patients at the GRU-3 hospital unit harbouring D34 ORF6 variant or WT, the small number of patients may have hindered the observation of an increased virulence potential of the deletion variant. In addition, the patient infected by the D26 variant is omitted from our analysis as no clinical data was available. Finally, we could not validate the enhanced expression of inflammatory cytokines in patients as plasma samples were not available in this retrospective cohort.

Taken together, these findings suggest that ORF6 deletion variants could play a major role in the inflammatory host-response, without impacting virus replication. Our study underlines the need to investigate how ORF6 deletions can impact host-response and clinical outcome, particularly since whole genome sequence analysis on the CoV-GLUE database has revealed converging clusters of similar ORF6 deletions mainly in Utah, USA and England, UK. Additional genomic and structural investigations are needed to explore the impact of ORF6 deletions, in terms of ribosomal frameshift stimulators, RNA translation production ratios, innate host immunity modulation, and clinical outcomes. The integration of more fundamental research dedicated to elucidating the factors that impact SARS-CoV-2 replication, transmission, and disease progression will ultimately help translational projects to advance the fight against the current COVID-19 pandemic.

## Supplementary Material

Supplemental Material

TEMI20201518_SupplementaryFigures_201209.docx

TEMI20201518_SupplementaryTable1_201209.xlsx
