# Supplementary material for: Characterization of SARS-CoV-2 ORF6 deletion variants detected in a nosocomial cluster during routine genomic surveillance, Lyon, France
Source: Emerg Microbes Infect. 2021 Jan 27;10(1):167–77. doi: 10.1080/22221751.2021.1872351 (PMC7850418; doi:10.1080/22221751.2021.1872351)
Supplement: TEMI20201518_SupplementaryFigures_201209.docx [file TEMI_A_1872351_SM1220.docx]

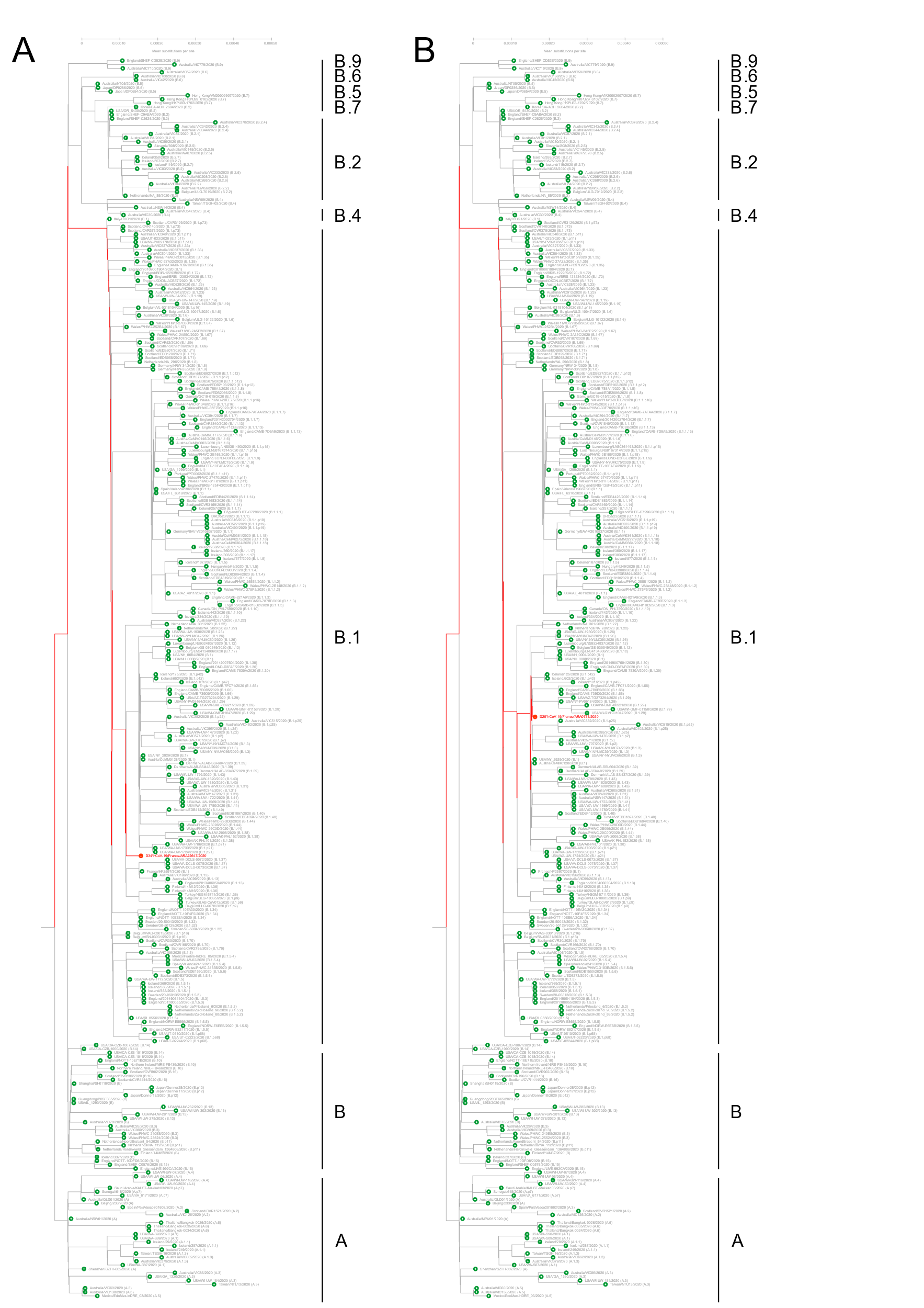


**SUPPLEMENTARY FIGURE 1**. CoV-GLUE phylogenomic placement map. The SARS-CoV-2 ORF6 D34 (A) and D26 (B) deletions are phylogenetically positioned against global SARS-CoV-2 sequences deposited on the GISAID database and annotated with PANGOLIN lineages. The tree was generated by the CoV-GLUE resource, which uses the RAxML (Randomized Axelerated Maximum Likelihood) software (Stamatakis, 2014). Relevant deletions are in red, while WT sequences are in green.

**
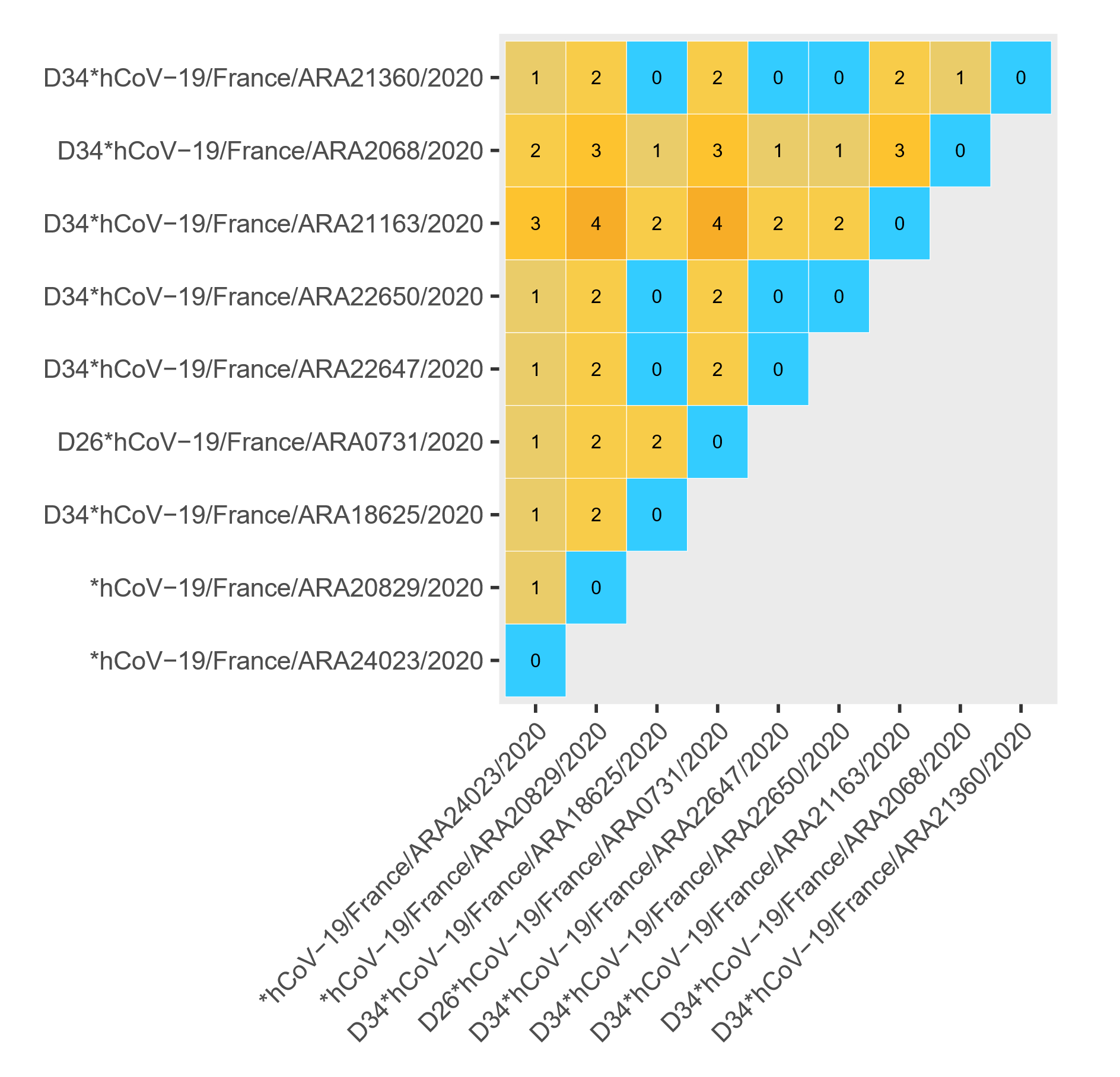
**

**SUPPLEMENTARY FIGURE 2**. Single nucleotide polymorphism heat map of SARS-CoV-2 ORF6 WT and deletion strains. Mismatch count between consensus sequences generated by each method compared 2 by 2 for each sample. Blue tiles correspond to perfect identity and orange tiles correspond to mismatches (number of mismatches is indicated inside the tile). Matrices were generated with an R script using Decipher (alignment), ape (distance matrices) and ggplot2 (charts) libraries. Of note, undetermined bases and deletions were not considered in the calculation of mismatches.

**SUPPLEMENTARY FIGURE 3**. *In vitro* immune-related gene transcriptomic profiles of WT SARS-CoV-2 and ORF6 deletion variants, D34 and D26. Infections were performed in triplicate on CaLu-3 cells at a multiplicity of infection of 0.2, and RNA was extracted 24 hours post-infection. The heatmap was generated by log2 Fold Change of gene expression (n=87 genes) against a mock infection after normalization with fours housekeeping genes (*DECR1, HPRT1, RPL19,* and *RPLP0*), evaluated by NanoString immune panel and nSolver analysis software.
